# A New Validated Liquid Chromatographic Method for the Determination of Loratadine and its Impurities

**DOI:** 10.3797/scipharm.1012-13

**Published:** 2011-02-12

**Authors:** Gajjela Ramulu, Yalavarthi Ravindra Kumar, Krishnamurthy Vyas, Mulukutla V. Suryanarayana, Khagga Mukkanti

**Affiliations:** 1 Analytical Research and Development, Integrated Product Development, Dr. Reddy’s Laboratories Ltd., Bachupally, Hyderabad-500072, India; 2 Center for Pharmaceutical Sciences, IST, J. N. T. University, Kukatpally, Hyderabad-500072, A.P, India

**Keywords:** Loratadine, Related impurities, Stability Indicating, RP-LC, Validation

## Abstract

An improved gradient, reversed-phase liquid chromatographic (RP-LC) method was developed and subsequently validated for the determination of Loratadine and its impurities/degradation products in pharmaceutical drug substance. Separation was achieved with Inertsil ODS-3V, 250 × 4.6 mm, 5μ column with gradient elution at a flow rate of 1.0 mL min^−1^. UV detection was performed at 220 nm. The described method is linear over a range of LOQ (0.044, 0.088, 0.084, and 0.072 μg mL^−1^ for impurity-B, impurity-C, impurity-D, and impurity-E respectively) to 1.2 μg mL^−1^ (0.6 μg mL^−1^ of the specification limit) for all the impurities and degradation products. The recovery of impurities were found to be in the range of 85–115 %. The method is simple, selective, and accurate for the quantification of impurities and degradation products of Loratadine in its bulk drug samples.

## Introduction

Loratadine is a second generation antihistamine drug closely structurally related to tricyclic antidepressants such as imipramine, and distantly related to the atypical antipsychotic quetiapine, used to treat allergies, which is available in the market as Claritin. Its chemical name is ethyl 4-(8-chloro-5,6-dihydro-11*H*-benzo[5,6]cyclohepta[1,2-*b*]pyridin-11-ylidene)-piperidine-1-carboxylate. Claritin is indicated for the relief of nasal and non-nasal symptoms of seasonal allergic rhinitis and for the treatment of chronic idiopathic urticaria. The pKa of loratadine is 5.0.

Patients with severe hepatic disorders may need to start with a lower dose. No dose adaptation is necessary for elderly or renally impaired patients Loratadine is usually compatible with breast-feeding (classified category L-2 by the American Academy of Pediatrics). In the U.S., it is classified as category B in pregnancy, meaning that animal reproduction studies have failed to demonstrate a risk to the fetus and there are no adequate and well-controlled studies in pregnant women.

Loratadine is given orally, is well absorbed from the gastrointestinal tract, and has rapid first-pass hepatic metabolism; it is metabolized by isoenzymes of the cytochrome P450 system, including CYP3A4, CYP2D6, and, to a lesser extent, several others. Loratadine is almost totally (97–99%) bound to plasma proteins. Its metabolite desloratadine is largely responsible for the antihistaminergic effects. It binds to plasma proteins by 73–76%.

Loratadine peak effect occurs in 1–2 hours, and its biological half-life is on average 8 hours (range 3–20 hours) with desloratadine half-life being 28 hours (range 9–92 hours), accounting for its long-lasting effect. About 40% is excreted as conjugated metabolites into the urine, and a similar amount is excreted into the feces. Traces of unmetabolized loratadine can be found in the urine [1].

**Fig. 1.**
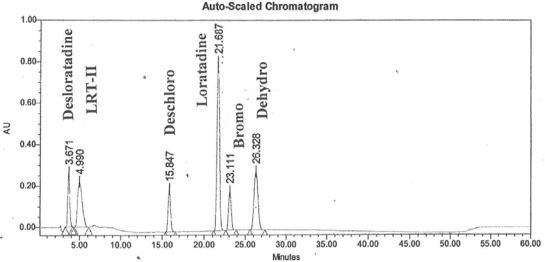
200 μg mL^−1^ of Desloratadine, LRT-II, Deschloro, Bromo, Dehydro impurities and 400 μg mL^−1^ of Loratadine.

Here, we present an improved analytical method for determination of impurities and degradation products in Loratadine drug substance, which will serve as a linear and accurate for its determination. A zypical chromatogram of all spiked impurities with loratadine is displayed in Figure 1. In the method, developed, herein, all the impurities and degradation products were well separated from the Loratadine peak. This method has been thoroughly validated as per the ICH guidelines.

## Results and Discussion

### Method development

In order to develop an improved suitable and robust LC method for the determination of Loratadine and its impurities and degradation products, different mobile phases and columns were employed to achieve the best separation and resolution. Initially, the isocratic method was used to estimation of impurities in Loratadine. Buffer (0.05 M monobasic potassium phosphate), Acetonitrile, Methanol and Triethyl amine (38:45:17:0.5 v/v) adjusted with ortho phosphoric acid to a pH of 3.6. Inertsil ODS-3V, 250 × 4.6 mm, 5μ was used for estimating the impurities. Separation was not observed between Impurity-B with one of the degradent i.e Impurity-A at about a retention time of 3.2 minutes. In addition, less resolution was observed between Impurity-E and Loratadine. Then pH increased to slightly 3.2 to 5.0 even though separation was not observed, again increased the pH to 6.9 separation was observed between Impurity-B and Impurity-A with 2.5 resolution but Impurity-E was merged with Loratadine. Thus there is a need to develop a robust method to quantify the Impurity-A and impurity-B in Loratadine. Several trials were made by using different mobile phase ratios, gradient programmes by varying buffer pH between 2 and 8 with C8 and C18 stationery phases. Based on the experimental trials it is understood that pH is playing the critical role in the separation between four impurities and Loratadine. Established LOD and LOQ for Impurity-A is 0.007% and 0.025% respectively in this method so it can also useful for the determination and identification of this impurity.

Very few methods appeared in the literature for the determination of Loratadine individually based on high-performance liquid chromatography (HPLC) [2–7].

Finally, the mobile phase-A contained buffer (0.05 M monobasic potassium phosphate), Acetonitrile, Methanol and Triethyl amine (38:45:17:0.5 *v/v*) adjusted with ortho phosphoric acid to a pH of 6.9 and mobile phase-B consisted buffer (0.05 M monobasic potassium phosphate), Acetonitrile, Methanol and Triethyl amine (38:45:17:0.5 *v/v*) adjusted with ortho phosphoric acid to a pH of 3.6.The flow rate of the mobile phase was 1.0 mL min^−1^.

### Analytical parameters and validation

After satisfactory development of method it was subject to method validation as per ICH guideline [8, 9]. The method was validated to demonstrate that it is suitable for its intended purpose by the standard procedure to evaluate adequate validation characteristics (system suitability, accuracy, precision, linearity, robustness, ruggedness, solution stability, LOD and LOQ and stability indicating capability).

### Precision

The precision of the determination of the impurities was checked by injecting six individual preparations of (400 μg mL^−1^) Loratadine spiked with 0.6 μg mL^−1^ of impurity-B, impurity-C, impurity-D and impurity-E and calculating the % RSD of % area for each compound. The intermediate precision of the method was also evaluated using different day and different instrument in the same laboratory are shown in Table 3 and 4.

### Limit of detection and Limit of quantification

Sensitivity was determined by establishing the limit of detection (LOD) and limit of quantification (LOQ) for impurity-B, impurity-C, impurity-D and impurity-E estimated at a signal to noise ratio of 3:1 and 10:1 respectively, by injecting a series of dilute solutions with known concentration. The limit of detection of a compound is defined as the lowest concentration that can be detected. LOD values were found to be 0.016, 0.028, 0.024, and 0.020μg mL^−1^ for impurity-B, impurity-C, impurity-D, and impurity-E respectively. The limit of quantification is the lowest concentration of a compound that can be quantified with acceptable precision and accuracy. LOQ values were found to be 0.044, 0.088, 0.084, and 0.072 μg mL^−1^ for impurity-B, impurity-C, impurity-D, and impurity-E respectively. The precision study was also carried out at the LOQ level by injecting six individual preparations of impurity-B, impurity-C, impurity-D, and impurity-E and calculating the % RSD for the areas of each impurity are shown in Table 5.

### Linearity

Standard solutions at eight different concentration levels ranging from LOQ (0.044, 0.088, 0.084, and 0.072 μg mL^−1^ for impurity-B, impurity-C, impurity-D, and impurity-E respectively) to 1.2 μg mL^−1^ (0.60 μg mL^−1^ of specification limit) were prepared and analyzed in order to demonstrate the linearity for all the impurities. Linearity regression analysis demonstrated acceptability of the method for quantitative determination range of LOQ (0.044, 0.088, 0.084, and 0.072 μg mL^−1^ for impurity-B, impurity-C, impurity-D, and impurity-E respectively) to 1.2 μg mL^−1^ of specification limit. The coefficient of correlation was found to be more than 0.995. The linearity results are shown in Table 6a and 6b.

### Accuracy

Accuracy of the method was demonstrated at four different concentration levels in triplicate. The analysis carried out at 0.30, 0.45, 0.60 and 0.90 μg mL^−1^ of specification limit. The mean recoveries of all the impurities were found to be in the range of 85–115 % as shown in Table 7. Typical chromatogram for all spiked impurities at 0.30, 0.45, 0.60 and 0.90 μg mL^−1^ with Loratadine is displayed in Figure 2, 3, 4 and 5 respectively.

### Robustness

In order to demonstrate the robustness of the method, system suitability parameters were verified by making deliberate changes in the chromatographic conditions, viz, change in flow rate from 1 mL min^−1^ (Figure 3a) to 0.8 and 1.2 mL min^−1^ (Figure 3b and 3c), change in pH of the buffer +0.2 unit, change in column temperature from 40°C (Figure 4a) to 35°C and 45°C (Figure 4b and 4c) and change the organic phase composition in the mobile phase from 100% (Figure 5a) to 90% and 110% (Figure 5b and 5c). The method was demonstrated to be robust over an acceptable working range of its HPLC operational parameters.

## Experimental

### Reagents and Materials

Samples of Loratadine and its related substances were received from process development laboratory of Dr. Reddy’s Laboratories Ltd., IPDO, Hyderabad, India. HPLC grade Acetonitrile, Methanol, monobasic potassium phosphate, ortho Phosphoric acid and Triethyl amine purchased from Merck, Germany., purchased from Regis technologies Inc, USA and high pure water was prepared by using Millipore Milli Q plus purification system.

### Equipment

The LC system used for method development and method validation was Waters LC system with a diode array detector (Model, quaternary gradient). The out put signal was monitored and processed using waters millennium software 3.2 version and released is March 31 2003.

### Chromatographic system

The chromatographic column used was Inertsil ODS-3V, 250 × 4.6 mm, 5μ. The mobile phase A contained buffer (0.05 M monobasic potassium phosphate), Acetonitrile, Methanol and Triethyl amine (38:45:17:0.5 v/v) adjusted with ortho phosphoric acid to a pH of 6.9 and mobile phase B consisted buffer (0.05 M monobasic potassium phosphate), Acetonitrile, Methanol and Triethyl amine (38:45:17:0.5 v/v) adjusted with ortho phosphoric acid to a pH of 3.6.The flow rate of the mobile phase was 1.0 mL min^−1^. The LC gradient program (Table 8) was set as: time (min)/% mobile phase- B: 0.01/0, 5/0, 9/20, 13/40, 17/70, 20/90, 25/100, 30/100, 35/70, 40/50, 45/20, 50/0 and 60/0. The column temperature was maintained at 40°C and the detection was monitored at a wavelength of 220 nm. The injection volume was 50 μL. Mobile phase A was used as diluent for sample preparations.

### Sample solution preparation

Weigh accurately 40mg of Loratadine sample into a 100mL volumetric flask, add 50mL of diluent and sonicate for 5minutes. Dilute to volume with diluent.

### Method validation

The proposed method was validated as per ICH guidelines [8, 9].

### System suitability

System suitability parameters were measured so as to verify the system performance. In the system suitability solution chromatogram resolution between impurity-D and Loratadine was measured. In the standard preparation theoretical plates for Loratadine peak was measured. Tailing factor for the Loratadine peak in standard preparation was measured. This all system suitability parameters covered the system, method and column performance.

### Precision

The precision of the determination of the impurities were checked by injecting six individual preparations of (400 μg mL^−1^) Loratadine spiked with 0.60 μg mL^−1^ of Impurity-B, Impurity-C, Impurity-D and Impurity-E and calculating the % RSD of area for each compound. The intermediate precision of the method was also evaluated using different analysts and a different instrument in the same laboratory.

### Accuracy

Accuracy of the determination of the impurities were carried out in triplicate at 0.30, 0.45, 0.60 and 0.90 μg mL^−1^ of the Loratadine concentration (400 μg mL^−1^).The percentages recoveries for the impurities were calculated.

### Limit of detection (LOD) and Limit of quantification (LOQ)

The LOD and LOQ for Impurity-A, Impurity-B, Impurity-C, Impurity-D and Impurity-E were estimated at a S/N of 3:1 and 10:1 respectively, by injecting a series of dilute solutions with known concentrations. Precision study was also carried at the LOQ level by injecting six individual preparations of Impurity-B, Impurity-C, Impurity-D and Impurity-E and calculated the %RSD for the areas.

### Linearity

Linearity of test solutions was prepared from stock solution at eight concentration levels from LOQ (0.044, 0.088, 0.084 and 0.072 μg mL^−1^ for impurity-B, impurity-C, impurity-D, and impurity-E respectively) to 1.20 μg mL^−1^ of analyte concentration. The peak area versus concentration data were subjected to least-squares linear regression analysis. The calibration curve was drawn by plotting impurities areas injections against the concentration expressed in percentage.

### Robustness

To determine robustness, experimental conditions were purposely altered and the tailing and theoretical plates for Loratadine peak was evaluated.

To study the effect of flow rate on the tailing and theoretical plates for Loratadine peak, it was changed from 1 mL min^−1^ to 0.8 and 1.2 mL min^−1^ (Refer figure 3a, 3b, 3c). The effect of pH on the tailing and theoretical plates for Loratadine peak of the impurities was also studied by varying the pH of mobile phase-A from 6.7 to 7.1.The effect of column temperature on the tailing and theoretical plates for Loratadine peak, it was changed from 40°C to 35°C and 45°C (Refer figure 4a, 4b, 4c). In all the above conditions, the components of the mobile phase were held constant.

## Conclusion

The present paper describes the development of a new HPLC method for the determination of impurities in Loratadine drug substance and its validation. The method was found to be selective, sensitive, precise and accurate for the determination of impurities and degradation products. This method can be used for the routine determinations in pharmaceutical quality control laboratories.

## Figures and Tables

**Fig. 2a:**
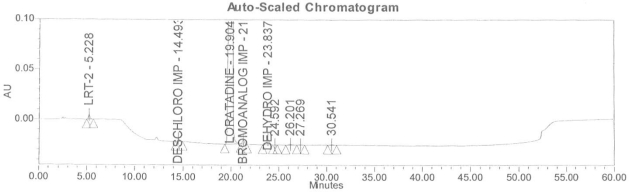
Typical chromatogram for 0.30 μg mL^−1^ of impurity-B, impurity-C, impurity-D and impurity-E spiked with Loratadine.

**Fig. 2b:**
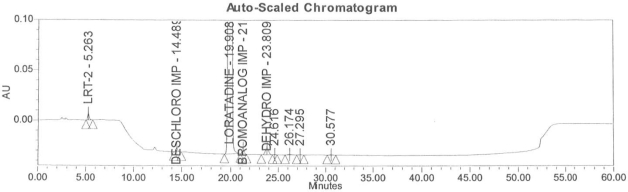
Typical chromatogram for 0.45 μg mL^−1^ of impurity-B, impurity-C, impurity-D and impurity-E spiked with Loratadine.

**Fig. 2c:**
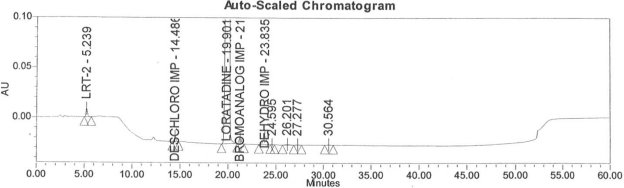
Typical chromatogram for 0.60 μg mL^−1^ of impurity-B, impurity-C, impurity-D and impurity-E spiked with Loratadine.

**Fig. 2d:**
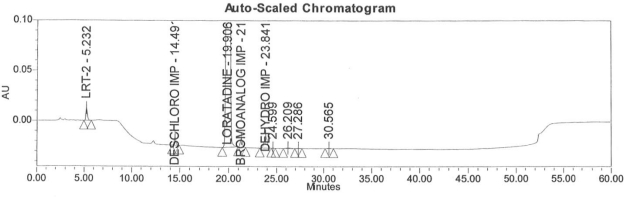
Typical chromatogram for 0.90 μg mL^−1^ of impurity-B, impurity-C, impurity-D and impurity-E spiked with Loratadine.

**Fig. 3a:**
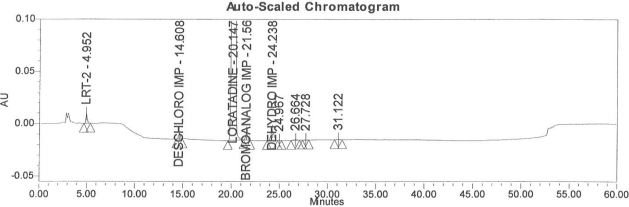
Typical spiked chromatogram for flow rate at 1 mL min^−1^.

**Fig. 3b:**
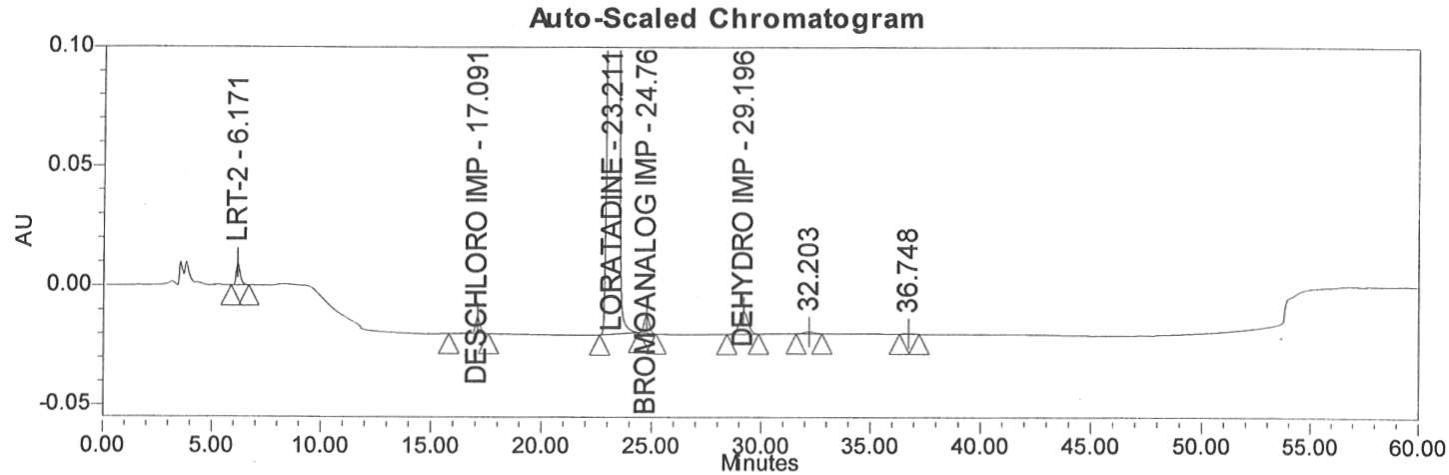
Typical spiked chromatogram for flow rate at 0.8 mL min^−1^.

**Fig. 3c:**
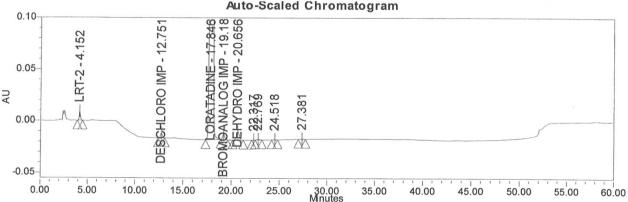
Typical spiked chromatogram for flow rate at 1.2 mL min^−1^.

**Fig. 4a:**
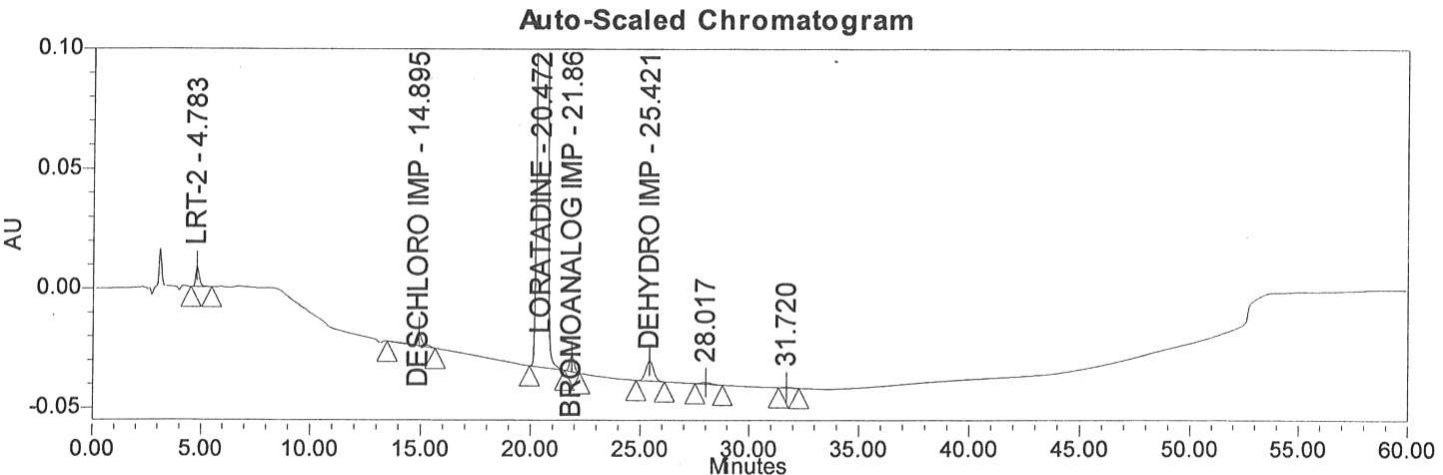
Typical spiked chromatogram for temperature at 40°C.

**Fig. 4b:**
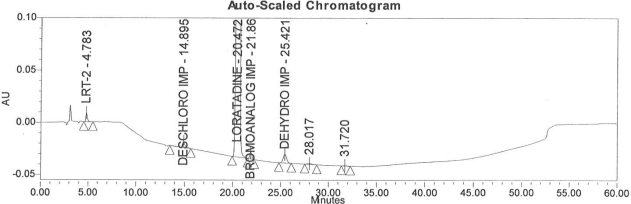
Typical spiked chromatogram for temperature at 35°C.

**Fig. 4c:**
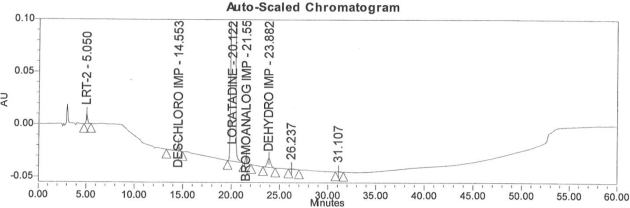
Typical spiked chromatogram for temperature at 45°C.

**Fig. 5a:**
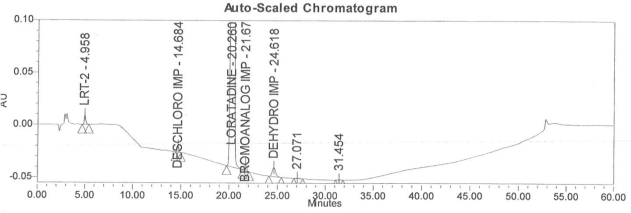
Typical spiked chromatogram for organic phase at 100%.

**Fig. 5b:**
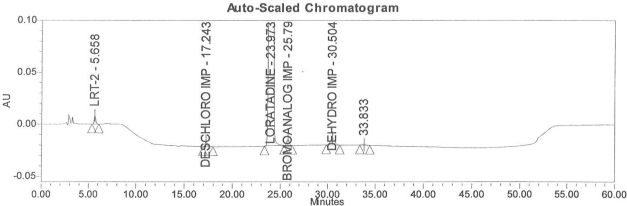
Typical spiked chromatogram for organic phase at 90%.

**Fig. 5c:**
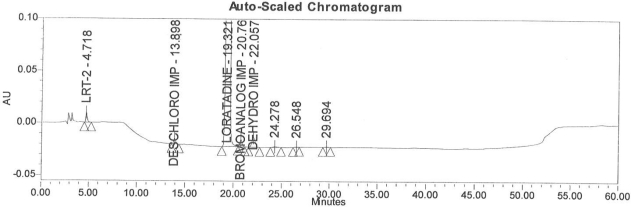
Typical spiked chromatogram for organic phase at 110%.

**Tab. 1.** Name of impurity, chemical structure and chemical name of Loratadine and five impurities (Desloratadine, LRT-II, Deschloro, Bromo, Dehydro)

**S.No.**	**Name of impurity**	**Structure**	**IUPAC Name**
1	Loratadine	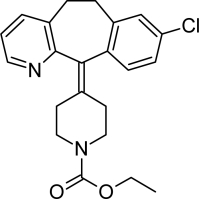	Ethyl 4-(8-chloro-5,6-dihydro-11*H-*benzo[5,6]cyclohepta[1,2-*b*]pyridin-11-ylidene)piperidine-1-carboxylate
2	Impurity-A (Desloratadine)	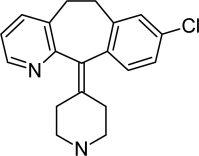	8-Chloro-11-piperidin-4-ylidene-6,11-dihydro-5*H*-benzo[5,6]cyclohepta[1,2-*b*]pyridine
3	Impurity-B (LRT-2)	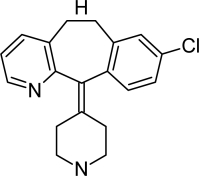	8-Chloro-11-(1-methylpiperidin-4-ylidene)-6,11-dihydro-5*H*-benzo[5,6]cyclohepta[1,2-*b*]pyridine
4	Impurity-C (Deschloro)	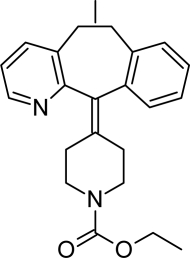	Ethyl 4-(5,6-dihydro-11*H*-benzo[5,6]cyclohepta[1,2-*b*]pyridin-11-ylidene)piperidine-1-carboxylate
5	Impurity-D (Bromo)	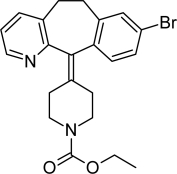	Ethyl 4-(8-bromo-5,6-dihydro-11*H-*benzo[5,6]cyclohepta[1,2-*b*]pyridin-11-ylidene)piperidine-1-carboxylate
6	Impurity-E (Dehydro)	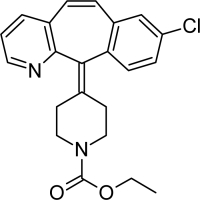	Ethyl 4-(8-chloro-11*H*-benzo[5,6]cyclohepta[1,2-*b*]pyridin-11-ylidene)piperidine-1-carboxylate

**Tab. 2.** Working concentration (% and μg mL^−1^) of Loratadine impurities and related retention time (RRT) with respect to Loratadine are as follow.

**Impurities**	**Working concentration**		**RRT**
	**μg mL^−1^**	**%**	
Impurity-A	0.6	0.15	0.16
Impurity-B	0.6	0.15	0.23
Impurity-C	0.6	0.15	0.73
Impurity-D	0.6	0.15	1.06
Impurity-E	0.6	0.15	1.21

**Tab. 3.** Results of method precision

**Preparation**	**Impurity-B**	**Impurity-C**	**Impurity-D**	**Impurity-E**
Prep-1	0.16	0.11	0.16	0.34
Prep-2	0.17	0.12	0.17	0.35
Prep-3	0.16	0.11	0.16	0.35
Prep-4	0.16	0.11	0.16	0.34
Prep-5	0.16	0.11	0.16	0.34
Prep-6	0.16	0.11	0.16	0.35
%RSD	1.4	1.5	0.5	2.3

**Tab. 4.** Results of intermediate method precision

**Preparation**	**Impurity-B**	**Impurity-C**	**Impurity-D**	**Impurity-E**
Prep-1	0.16	0.11	0.16	0.34
Prep-2	0.17	0.12	0.17	0.35
Prep-3	0.16	0.11	0.16	0.35
Prep-4	0.16	0.11	0.16	0.34
Prep-5	0.16	0.11	0.16	0.34
Prep-6	0.16	0.11	0.16	0.35
%RSD	2.5	3.6	2.5	1.5

**Tab. 5.** Results of LOQ precision

**Preparation**	**Impurity-B**	**Impurity-C**	**Impurity-D**	**Impurity-E**
Prep-1	15812	11794	14659	26316
Prep-2	13658	12441	13550	25596
Prep-3	13969	12384	13770	26156
Prep-4	16232	12115	14672	26484
Prep-5	13995	12389	14635	25672
Prep-6	14087	12391	13657	25900
%RSD	7.5	2.1	3.9	1.4

**Tab. 6a.** Linearity results

**Conc. μg**	**Impurity-B**	**Impurity-C**	**Impurity-D**	**Impurity-E**
LOQ	16359	12229	36205	64190
0.15	37524	24050	47599	100585
0.30	54121	37833	61747	132200
0.45	82057	54758	79606	174929
0.60	108804	68981	93864	205192
0.75	127788	87254	112811	249905
0.90	150620	102170	128301	282397
1.20	189488	136298	165058	375571
**R^2^**	**0.998**	**0.999**	**0.999**	**0.998**

LOQ values were 0.044, 0.088, 0.084 and 0.072 μg mL^−1^ for impurity-B, impurity-C,

**Tab. 6b.** Correlation coefficient (R^2^), slope and y-intercept results for impurity-B, impurity-C, impurity-D, and impurity-E.

	**Impurity-B**	**Impurity-C**	**Impurity-D**	**Impurity-E**
R^2^	0.998	0.999	0.999	0.998
Slope	150956.4	108938.6	112880	264570.9
Y-Intercept	12932.3	5013.0	28085.1	51879.6

**Tab. 7.** Accuracy results

**Conc. μg mL^−1^**	**Impurity-B(%)**	**Impurity-C(%)**	**Impurity-D(%)**	**Impurity-E(%)**
0.30	100.6	101.1	102.6	100.1
0.45	101.1	101.0	102.5	103.5
0.60	99.7	100.7	100.7	100.7
0.90	99.5	99.8	100.6	100.2

**Tab. 8.** Gradient program

**Time(min)**	**% Mobile phase-A**	**% Mobile phase-B**	**Gradient line**
0.00	100.0	0.0	6
5.00	100.0	0.0	6
9.00	80.0	20.0	6
13.00	60.0	40.0	6
17.00	30.0	70.0	6
20.00	10.0	90.0	6
25.00	0.0	100.0	6
30.00	0.0	100.0	6
35.00	30.0	70.0	6
40.00	50.0	50.0	6
45.00	80.0	20.0	6
50.00	100.0	0.0	6
60.00	100.0	0.0	6
